# PROPOSAL OF A REVISIONAL SURGERY TO TREAT SEVERE NUTRITIONAL DEFICIENCY POST-GASTRIC BYPASS

**DOI:** 10.1590/0102-6720201600S10024

**Published:** 2016

**Authors:** José SAMPAIO-NETO, Alcides José BRANCO-FILHO, Luis Sérgio NASSIF, André Thá NASSIF, Flávia David João De MASI, Graciany GASPERIN

**Affiliations:** Bariatric Surgical Service, Santa Casa de Misericórdia Hospital, Curitiba, PR, Brasil.

**Keywords:** Bariatric surgery, nutritional deficiency, Roux-en-Y gastric bypass, Gastroplasty

## Abstract

**Background:**

The gastric bypass has nutritional and electrolyte disturbances rate of approximately 17%. The most common deficits are protein malnutrition, ferric and zinc, in addition to the vitamin. Although rare, some malnutrition stages reach such severity that ends up being necessary hospitalization and sometimes revisional or reversal surgical procedures.

**Aim:**

To present a proposal of surgical revision for treatment of severe malnutrition after bariatric surgery.

**Methods:**

The procedure is to reconstitute the food transit through the duodenum and proximal jejunum, keeping the gastric bypass restrictive component. As an additional strategy, the gastric fundus resection is performed, aiming to intensify the suppression of the greline and avoiding excessive weight regain.

**Results:**

After initial stabilization, nutritional and electrolytic support, the procedure was performed in two patients as definitive treatment of malnutrition status. Good results were observed at one year follow up.

**Conclusion:**

As improvement option and/or resolution of the nutritional alterations, surgical therapy is one of the alternatives. There is still no consensus on the surgical technique to be performed. This procedure is based on pathophysiological factors for the treatment of this condition, with good initial results, without significant clinical alterations. Longer follow-up will determine its effectiveness.

## RESUMO

**Racional:**

O bypass gástrico consta com taxa de distúrbios nutricionais e eletrolíticos de aproximadamente 17%. Os déficits mais frequentes são a desnutrição proteica, férrica e de zinco, além das vitamínicas. Apesar de raros, alguns quadros de desnutrição atingem tal gravidade que acaba sendo indicada internação e, por vezes, procedimentos cirúrgicos revisionais ou de reversão.

**Objetivo:**

Apresentar proposta de cirurgia revisional para tratamento de desnutrição severa após bypass gástrico.

**Métodos:**

O procedimento consiste em reconstituir o trânsito alimentar pelo duodeno e jejuno proximal, mantendo o componente restritivo do bypass gástrico. Como estratégia adicional, é realizada ressecção do fundo gástrico, visando intensificar a supressão da grelina e evitando reganho excessivo de peso.

**Resultado:**

Após estabilização inicial com suporte hidroeletrlítico e nutricional, o procedimento foi realizado em dois pacientes como tratamento definitivo do quadro de desnutrição. Bons resultados foram observados em seguimento de um ano.

**Conclusão:**

Como opção de melhora e/ou resolução da defasagem nutricional, a terapia cirúrgica é uma das alternativas. Ainda não há consenso quanto à técnica a ser utilizada. O procedimento aqui apresentado é baseado em fatores fisiopatológicos para o tratamento desta condição, com bons resultados iniciais, sem efeitos colaterais significativos. Seguimento de mais longo prazo é necessário para determinação de sua eficácia.

## INTRODUCTION

Initially used only as an alternative for the treatment of metabolic disorders, bariatric surgery have more and more space also for the treatment of morbid obesity, being a safe and effective procedure^23^. Today, many techniques are available, but the Roux-en-Y gastric bypass among them is the most performed procedure not only in Brazil but all over the world. The Roux-en-Y gastric bypass is a restrictive-malabsorptive technique that promotes early satiety due to gastric reduction, but has long known complication due to food restriction and malabsorption in relation to gastric reduction and duodenum manipulation, restricting the intestinal space in which the food digestive juices mix happens^4^.

Malabsorption associated with a deficient caloric intake after surgery and fast weight loss results in a remarkable rate of nutritional and electrolyte disturbances. The most common disorders are albumin, hemoglobin, iron, zinc and vitamins, especially vitamin B12 and D3^10^. Each of these components has action on a specific function and optimal pH depending on the production or absorption of hormones, proteins, among others substances which occur in the small intestine or stomach, but modified by surgical technique^7^. The body has reserves that can mask vitamin deficiency over a period which can range from days to years after surgery. In addition, many patients are admitted to the procedure already with nutritional deficiencies, which can aggravate the situation in the postoperative period^6^.

Secondary to this vitamin and mineral deficits occur diarrhea and possible development of other conditions such as peripheral neuropathies and visual damage by vitamin deficiency, bone loss by limiting the absorption of calcium and vitamin D, anemia due to lack of iron and proteins and also the dumping syndrome^10^.

The clinical trial of reversal of these deficits is given mainly by the use of multivitamins, but adherence to this kind of treatment is flawed because it depends on continuity and must be unanimous. Finally, some malnutrition boards reach such severity that the reversal surgery ends up being indicated^7^.

The aim of this study was to present surgical technique of gastroplasty partial reversal as surgical treatment option for severe malnutrition correction after gastric bypass.

## METHOD

The study was approved by the Ethics Research Committee of Santa Casa Hospital, Curitiba, PR, Brazil. Patients signed the free and informed consent form for the surgery.

**FIGURE 1:**
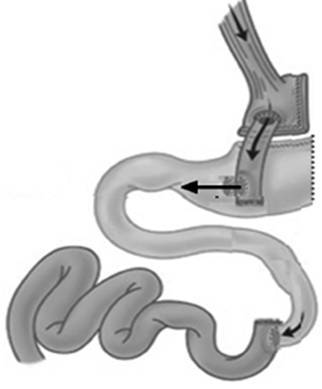
Proposal for a revisional procedure for treating severe nutritional deficiency after bariatric surgery

The procedure consists on open surgery, with access from the midline followed by release of supramesocolic adhesions. After identification of the bypassed stomach, it is held release of the vessels of greater curvature, followed by resection of the background and part of the gastric body with electrocautery and strengthening clamps line with manual suture. Then, is proceeded side anastomosis between food loop in 10 cm from the previous gastroenterostomy and gastric antrum at 4 cm from the pylorus. After section of the feed loop distally to the new anastomosis, the original enteroanastomosis is resected, with control of the underlying mesentery (Figure 1). Finally, negative methylene blue test is carried out ending the procedure.

## RESULTS

This procedure was applied in two cases in which revisional surgery was indicated as malnutrition treatment after stabilization of severe undernutrition and electrolyte imbalance. The patients were women between the fourth and fifth decade of life, prior submitted to laparotomic Roux-enY gastric bypass; in that time they were with BMI 38.61 and 51.92. They were re-hospitalized two years later malnutrition with hypoalbuminemia, anasarca, diarrhea, BMI 19.9 and BMI 19.06, with severe asthenia, malnutrition, diarrhea, giving the suspection of short common loop. 

The first patient was initially treated with partial parenteral nutrition, and subsequently by total. Without improvement, revisional surgery was indicated due protein deficiency, altering to 370 cm the common handle and adding gastrostomy (G). After two months, was carried out partial reversal procedure (PR). With two days of the procedure, diet was changed to liquid. Twenty days after as outpatient clinic, the patient was asymptomatic with improvement in diarrhea and anasarca. Denied food intolerance, good intake of legumes and vegetables, one to two bowel movements daily (Bristol 4), making use of multivitamin and with BMI 26.67, complaining of joint pains (^Figure1^). 

**FIGURE 2:**
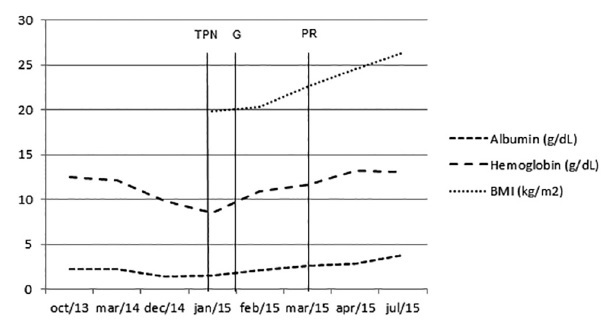
Case 1 postoperative monitoring

The second case had hypertension controlled via drug therapy before gastric bypass in Roux-en-Y. After, blood pressure was normal with liberation of the use of anti-hypertensive. As outpatient, had malnutrition symptoms, accompanied by recurrence of hypertension and depression requiring drug treatment. In reoperation, common loop measured 280 cm. She received blood transfusions, plasma and total parenteral nutrition (TPN) preoperatively and underwent surgery to lengthen food loop to 370 cm. After three months, she was with BMI 20.69, the same malabsorptive syndrome, anasarca, anemia, hypoalbuminemia and suspected incretinic hyperactivity; she was submitted to gastrostomy (G) and released for treatment at home with improved of hypoalbuminemia and depression. After 12 months, BMI was 25.15,bur maintained malnutrition and intestinal malabsorption and was indicated partial reversal (PR) surgery. After four months postoperatively as outpatient the BMI was 32.4, PEE 74.06%, free diet, good acceptance of legumes and vegetables, once or twice daily evacuation and proper hydration. Referred heartburn and dumping complaints with sweets and fried food (Figure 2).

**FIGURE 3:**
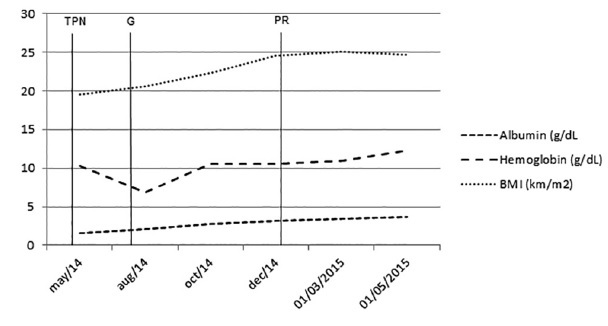
Case 2 postoperative monitoring

## DISCUSSION

The basis for the reduction and weight maintenance is to restrict food intake and/or malabsorption of nutrients, as well as decrease the gastrointestinal transit time, which can provide many nutritional deficiencies. However, the majority of candidates for bariatric surgery already have deficient micronutrients even before the surgical procedure, and that preoperative disability associated with the anatomical and physiological changes caused by surgical technique may predispose to the development of severe vitamin and mineral deficiencies^7^.

Regarding the surgical technique, Roux-en-Y gastric bypass reduces stomach volume to approximately 30-50 ml. To do so, is required to delete gastric fundus, body and antrum, as well as duodenal portion of the small intestine. These areas have specific function producing substances necessary for the absorption of micronutrients, such as intrinsic factor for activation of vitamin B12, HCl for reducing Fe^3+^ to Fe^2+^ at the gastric body and the absorption of iron, calcium, thiamine, magnesium and folic acid (vitamin M) in the duodenum. However, the most common nutritional deficiencies regarding this technique are vitamin B12, iron and folic acid^24^. Besides the deficiency of micronutrients, still occurs deficiency of macronutrients as the protein deficit^2^_._

Among the causes, there is excessive food restriction due to decreased gastric volume. Surgical manipulation of the gastrointestinal tract associated with drastic change in eating habits trigger a change in bowel habits, which leads to diarrhea, constipation or flatulence after surgery. It has been in diarrhea a major cause of malnutrition. The most common causes of post bypass diarrhea are intolerance to bile salts, exacerbation of previously subclinical lactose intolerance, irritable bowel syndrome, malabsorption and a long loop bypass^15^. Among other causes are dumping syndrome, infectious origin, ileocecal valve dysfunction and pelvic diseases^1^. 

Another significant cause of malnutrition is short bowel syndrome after bypass; however, nowadays it is recommended to throw it away because of the small number of patients who actually have this situation when submitted to loop approach. This situation leads to malabsorptive state due to massive alteration of the small intestine promoted by the bypass, that may result in intestinal failure, dehydration and malnutrition without oral or intravenous supplementation.

The incretinic hyperactivity, among the above causes, comes as a diagnosis of exclusion. In this situation, nutritional deficiency occurs with early stimulation of the distal gut, increased secretion of GLP- 1 and PYY, and hyperinsulinemia and intestinal hypermotility. 

The specific clinical signs are noticeable only in the more advanced stage of disability^16^. When symptomatic, primarily patients with disabilities have diarrhea and moderate dehydration. Among the complications has been the early and late ones, and within these two categories can find many clinical situations already exhaustively related^5^ ^,^ ^11^. Associated with metabolic complications are those of psychological origin^3^ ^,^ ^26^.

Monitoring of nutritionists after the procedure is necessary, aimed at modifying the diet and may reach the restriction of lactose consumption and eventually gluten, nutritional education based on limitations and needs from the surgical technique, and adequate fluid intake^2^ ^,^ ^15^. In addition, the professional cardiac and endocrinological monitoring is needed due to hypertensive continuous evaluation, lipid and insulin necessities, adjustment of drug doses to patients who need it, either by variation weight or comorbidities after surgery. In addition, it must also be the follow up made by other professionals that belong to the multidisciplinary team of bariatric care^13^.

Adjunct to these, there is a need for multivitamin and mineral supplementation^13^. However, the success of any clinical therapeutic method depends on adherence to treatment and return for follow-up and documentation of nutritional evolution. 

The proposed surgery is feasible and safe, based on the trigger mechanism of the syndrome; it has similar effect like the other reversal techniques, but with low levels of mortality. Performing partial gastrectomy in the excluded stomach, the gastric pouch and orexigenic stimulus of ghrelin is decreased, which prevent excessive weight regained. Anastomosing food loop with the pre-pyloric antrum restores normal intestinal tract and entero-insular axis, resulting in PYY and GLP 1 decrease and, consequently, intestinal hypermotility and hyperinsulinemia. It is possible to get further reversal of nutritional deficiency and hypoalbuminemia, giving a better quality of life and health status for these patients. However, patients in the sample had a sensitive weight regain.

## CONCLUSION

As improvement option and/or resolution of the nutritional alterations, surgical therapy is one of the alternatives. There is still no consensus regarding the surgical technique to be performed. This procedure is based on pathophysiological factors for the treatment of this condition, with good initial results, without significant clinical alterations. Longer follow-up will determine its effectiveness.
